# Utilization of urea and expression profiles of related genes in the dinoflagellate *Prorocentrum donghaiense*

**DOI:** 10.1371/journal.pone.0187837

**Published:** 2017-11-08

**Authors:** Xiaoli Jing, Senjie Lin, Huan Zhang, Claudia Koerting, Zhigang Yu

**Affiliations:** 1 College of Marine Life Science, Ocean University of China, Qingdao, China; 2 Department of Marine Sciences, University of Connecticut, Groton, United States of America; 3 Laboratory for Marine Ecology and Environmental Science, Qingdao National Laboratory for Marine Science and Technology, Qingdao, China; 4 Key Laboratory of Marine Chemical Theory and Technology, Ministry of Education, Qingdao, China; University of Cambridge, UNITED KINGDOM

## Abstract

Urea has been shown to contribute more than half of total nitrogen (N) required by phytoplankton in some estuaries and coastal waters and to provide a substantial portion of the N demand for many harmful algal blooms (HABs) of dinoflagellates. In this study, we investigated the physiological and transcriptional responses in *Prorocentrum donghaiense* to changes in nitrate and urea availability. We found that this species could efficiently utilize urea as sole N source and achieve comparable growth rate and photosynthesis capability as it did under nitrate. These physiological parameters were markedly lower in cultures grown under nitrate- or urea-limited conditions. *P*. *donghaiense* N content was similarly low under nitrate- or urea-limited culture condition, but was markedly higher under urea-replete condition than under nitrate-replete condition. Carbon (C) content was consistently elevated under N-limited condition. Consequently, the C:N ratio was as high as 21:1 under nitrate- or urea-limitation, but 7:1 under urea-replete condition and 9:1 to 10:1 under nitrate-replete condition. Using quantitative reverse transcription PCR, we investigated the expression pattern for four genes involved in N transport and assimilation. The results indicated that genes encoding nitrate transport, urea hydrolysis, and nickel transporter gene were sensitive to changes in general N nutrient availability whereas the urea transporter gene responded much more strongly to changes in urea concentration. Taken together, our study shows the high bioavailability of urea, its impact on C:N stoichiometry, and the sensitivity of urea transporter gene expression to urea availability.

## Introduction

Harmful algae blooms (HABs) are an important ecological phenomenon that pose serious impacts on ecosystems, economy, and public health and have been increasing globally [1]. Dinoflagellates, one of the most important groups of primary producers, are the most important contributors to HABs and algal toxins in the marine ecosystem [2]. One of the most important recognized drivers of HABs is excess nitrogen (N) nutrient. High abundances of N compounds are often related with the outbreaks of HABs caused by dinoflagellates [3,4]. N-nutrient occurs in various chemical forms, including the inorganic NO_3_^-^, NH_4_^+^ and the organic urea. The different forms of N may be utilized at different efficiencies in different species, allowing for the success and productivity of different phytoplankton species at different times [5]. Understanding the physiological and molecular mechanisms that govern N sensing and utilization in phytoplankton communities is pivotal for predicting their ecological success [6], especially the formation of HABs.

Among various forms of N nutrients in the ocean, ammonium is energetically preferable, but nitrate is the known major form available to phytoplankton. As demonstrated in field and laboratory experiments [7,8], however, the organic N form urea can also be utilized as sole N source by many species of phytoplankton. In some estuarine and coastal areas, urea contributes to over half of total N required by phytoplankton and provides a substantial portion of the N demand for many harmful algal blooms (HABs) of dinoflagellates [7] such as *Lingulodinium polyedrum* and *Alexandrium catenella* [9,10] as well as of other groups such as the pelagophyte *Aureococcus anophagefferens* [11–13].

N assimilation in marine phytoplankton is a tightly regulated process that fuels autotrophic and heterotrophic C metabolism. At the molecular level, this involves proteins responsible for the uptake and enzymes responsible for assimilation of the N nutrient. Some transporters are constitutively expressed whereas others are regulated by varied environmental factors such as light and abundances of substrates. As reported, dinoflagellates possess a full range of transporters for uptake and enzymes for assimilation of different forms of N; they are thus versatile in acquiring N nutrients [1,14]. This includes nitrate transporters (NRT), a plasma membrane protein that delivers nitrate to the cytosol, nitrate reductase, a cytosolic enzyme that reduces nitrate to nitrate, which is further reduced to ammonium by a nitrite reductase, a chloroplast enzyme. NRT encoding gene (*nrt*) has been identified in some dinoflagellates: *A*. *catenella*, *L*. *polyedrum*, and *Symbiodinium kawagutii* [15–18].

Urea transporters (UT) regulate urea transport and ureases catalyze the metabolism of urea to release ammonium and carbonate. Urease (URE), commonly distributed in various classes of algae [19], is an amidohydrolase that requires nickel (Ni) in the active site [20]. The role of Ni in urea assimilation is well established and historically has been used to explain the “nutrient-like” depth profile of Ni in seawater [21]. For many algal taxa, it has been shown that growth with urea as the sole N source is Ni-dependent [19]. Ni uptake is regulated by a high-affinity Ni transporter (NiT). Yet, few studies have focused on Ni transporters in phytoplankton. Overall, information concerning gene expression related to N nutrient transport and utilization in dinoflagellates is relatively limited.

In this study, we investigated the responses of NRT, UT, URE, and NiT in the harmful algal bloom forming dinoflagellate, *Prorocentrum donghaiense*, using quantitative reverse transcription PCR (RT-qPCR) to nitrate-replete, urea-replete, and their respective limited conditions. *P*. *donghaiense* is genetically very similar to *Prorocentrum dentatum* based on nuclear rRNA genes and mitochondrial genes [22], which is widely distributed in Europe, America, Australia, and New Zealand [23]. Dinoflagellates are known to regulate most of their genes (more than 70%) at post-transcriptional levels [2]. However, at least some of the genes that regulate uptake of phosphorus and nitrogen (N) nutrients are transcriptionally responsive to nutrient conditions [1,24–26], justifying the use of RT-qPCR to investigate response of N-nutrient related genes to different N-nutrient conditions. Meanwhile, we examined how changes in the N condition impacted cell growth, photosynthesis capacity and cellular content of C and N. We found that cells of this species could utilize urea as sole N source as efficiently as growing on nitrate. Limitation of nitrate or urea caused negative effects on physiological status and significant responses in the expression of these genes. Our study provided a baseline dataset for *P*. *donghaiense* and potential molecular markers for detecting urea utilization and generally N limitation.

## Methods

### Algal culture and experimental setup

*P*. *donghaiense* strain CCMAXU-364 (Center for Collections of Marine Algae in Xiamen University), isolated from East China Sea in 2009, and was grown in L1 medium. At 20 ± 1°C, experimental cultures were grown in 2 liters flasks with filtered (0.22 μm) and autoclaved Long Island Sound seawater (31 PSU) and incubated on a 14:10 h light: dark cycle with a photon flux of 100 μE m^-2^ s^-1^. N-limited and replete cultures were prepared in triplicates. The nitrate-replete condition was given in L1 medium, which served as a control. In the nitrate-limited cultures, the L1 was modified by changing from 882 μM to 11 μM starting nitrate. Urea-replete cultures were grown in L1 medium except that nitrate was replaced with urea at the same molar concentration (882 μM). In the urea-limited cultures the starting urea concentration was given only at 11 μM. The culture experiment lasted eight days. To conduct the experiments under axenic condition, a cocktail of antibiotics, including ampicillin, kanamycin monosulfate and streptomycin, was added to all inoculated cultures at the concentration of 50 mg L^-1^ each.

To monitor growth rate (μ), cell concentrations were monitored every other day using a Sedgewick-Rafter counting chamber (Phycotech, MI, USA). Growth rate was calculated using the formula: μ = ln (*N*_*2*_/*N*_*1*_)/(*t*_*2*_-*t*_*1*_), where *N*_*2*_ and *N*_*1*_ are total cells on day *t*_*2*_ and day *t*_*1*_, respectively. After keeping the samples in darkness (15 min), photochemical efficiency (Fv/Fm ratio) was quantified with 10-AU Flurometer (Turner, California, USA) following DCMU (3-(3,4-dichlorophenyl)-1,1-dimethylurea) protocol [27].

### Measurement of extracellular and intracellular nitrogen contents

At days 0, 2, 4, 6 and 8, nitrate concentration was measured in the nitrate-replete and nitrate-limited cultures whereas urea concentration was measured in the urea-replete and urea-limited cultures from 0.22 μm filtrates of 50 mL samples collected from the respective cultures. The filtrates were kept frozen at -80°C until subsequent analysis. Prior to nutrient analysis, samples were transferred into pre-cleaned sample vials (acid treated for 48 h first, then wrapped with aluminum foil and combusted at 450°C for 3–5 h in a Muffle Furnace). Nitrate concentration was analyzed on SmartChem^®^, a discrete nutrient auto-analyzer (Unity Scientific, Brookfield, CT, USA). The measurement was based on USEPA 353.2. Revision 2.0 and standard method 4500 NO_3_^-^ F 18^th^ and 19^th^ Editions [28] modified for SmartChem^®^, which gave total nitrate plus nitrite.

Urea analysis was performed on Shimadzu TOC-VCPN analyzer, following standard method 5310B and APHA (2005) methods [28], and Alvarez-Salgado and Miller (1998) [29]. Using the Shimadzu analyzer linked to the TNM-1 nitrogen analyzer, dissolved C and dissolved N contents of samples were analyzed using 720°C high temperature combustion and catalytic oxidation. The TNM-1 analyzer uses chemiluminescence (CLD) for detection of total dissolved N. Standard urea reference solutions (0, 50, 100, 200, 400, 600 and 800 μM) were used to determine a total N calibration curve. Cell-specific nitrogen uptake rates (V, pmol N cell^-1^ day^-1^) were estimated by dividing changes of extracellular nitrogen concentration (*C*) by the changes of cell concentrations (*N*) and days (*t*): V = (*C*_*2*_*-C*_*1*_)/(*N*_*2*_*-N*_*1*_)(*t*_*2*_*-t*_*1*_).

In the exponential growth stage (the 4^th^ and 6^th^ day), 1.7 × 10^6^ cells of each sample were collected onto 25-mm GF/F filter (pre-combusted at 450°C for 5 h) and combusted in Fisons NA 1500 series II elemental analyzer (Costech Analytical Technologies, Italy) following the EPA method 440 [30,31]; the weight of C and N elements were averaged to per cell content.

### Sampling, RNA extraction and qRT-PCR

When the experimental cultures were in exponential growth stage (6^th^ day), the cells were collected at 20°C by centrifugation; each cell pellet was then resuspended in 1 mL TRI-Reagent (Molecular Research Center Inc., OH, USA) and stored at -80°C until RNA extraction.

Total RNA was extracted following Trizol manufacturer’s instruction with some modification. The samples were homogenized by adding silica beads (0.5 mm diameter) to approximately the same volume of the cell pellet in a 2 mL tube, and vibrating the tube on MP Fast Prep-24 Tissue and Cell Homogenizer (MP Biomedicals, OH, USA) at 6 m s^-1^ for three cycles each for 1 min, cooled on ice for 1 min between cycles. The cell lysates were then centrifuged at 12,000 × g, 4°C for 4 min, the supernatant from each sample was removed to a clean 1.5 mL tube and left on ice. Next, 0.2 mL chloroform was added and the tube was shaken vigorously by hand for 15 sec, incubated at RT for 2 to 3 min, and then centrifuged at 12,000 × g for 15 min at 4°C. The aqueous layer on the top was removed into a fresh 1.5 mL tube and the volume was measured using a pipette. RNA was precipitated by adding 1/2 volume of isopropanol and 1/2 volume of Precipitation Solution (0.8 M sodium citrate and 1.2 M sodium chloride dissolved in DEPC-water, used to remove polysaccharide and glycoprotein contaminants in the RNA extract). After centrifugation at 12,000 × g, 4°C for 10 min, the RNA pellet was washed using 1mL 70% ethanol twice. Finally, after centrifugation at 12,000 × g, 4°C for 5 min, the RNA pellet was air-dried for 5 min at RT, and dissolved in 20 μL DEPC-water. Based on our prior experience, RNA so extracted contained enzyme inhibitors; therefore a further purification step was taken. 1 mL TRI-Reagent was added to each RNA sample, followed by 0.2 mL chloroform. After centrifugation at 12,000 × g, 4°C for 15 min, the aqueous phase was removed into a fresh tube and another wash with equal volume of phenol/ chloroform (v/v ratio of 5/2, pH 4.0) was conducted twice. Next, to the retrieved aqueous phase, an equal volume of 70% ethanol was added and mixed, and the mixture was loaded into a Qiagen RNeasy RNA column (Qiagen, CA, USA). Following the manufacturer’s instruction, further RNA purification and evolution was performed.

The concentration and quality of RNA were determined by NanoDrop-2000 Spectrophotometer (Thermo Scientific, Wilmington, DE, USA). Because RNA is rich in nitrogen and its cellular content may differ under different N-nutrient conditions, we calculated RNA content per cell by dividing total RNA yield by total cells number collected per sample. The 1st strand cDNA was synthesized using iScript Select cDNA synthesis kit (Bio-Rad, USA) with random primer and 100 ng total RNA of each sample.

The products of reverse transcription were used as templates for qRT-PCR using the SYBR Green SSO IT Supermix (Bio-Rad, USA). qRT-PCR were performed in 96-well plates on an ABI StepOne Real-Time PCR System (ABI, USA), with each reaction containing 250 nM of each primer, 1 μL cDNA template and 5 μL 2×SYBR Green Supermix in a total volume of 10 μL. Specific primers of the nitrate transporter (*PdNRT*), urea transporter (*PdUT*), urease (*PdURE*) and high-affinity nickel transport protein (*PdNiT*) were designed (Table 1) and their specificity was verified using regular PCR followed by electrophoresis. PCR amplification efficiencies (E) of each primer pair were calculated following E% = (10^−1/slope^– 1) × 100 [32], in which the slope were obtained from the standard curve generated from a serial 10-fold dilutions of a cDNA sample prepared by pooling an aliquot of each experimental RNA sample. From the same dilution series correlation coefficient (R^2^) was also calculated.

**Table 1:** Information of primers and thermal cycling conditions used in RT-qPCRs.

^a^Primer name	Gene name	^a^Sequences (5’-3’)	Product size (bp)	Annealing temperature	E%	R^2^
*PdCalm*	*Calmodulin*	F: AGTTCAAGGAGGCGTTCTCTTTGTTC R: CCATCAAGGACAAGAACTCGGGAAAG		62°C	96.1	0.995
*PdNRT*	*nitrate transporter*	F: AAGCTTTACGCGGGCTATGT R: AAAGGAGCCTGTGACTTGGG	172	62°C	95.8	0.989
*PdUT*	*Urea active transporter*	F: ATCTCGCCGAACTCAACCTG R: GGTGTACCAGTTCATGGGCA	168	62°C	95.6	0.999
*PdURE*	*Urease*	F: GCCTTTGATGCCAATGTCGG R: ACCTCCTCGCAAAGGTTGAG	197	60°C	95.5	0.984
*PdNiT*	*high-affinity nickel transport protein*	F: GGAGCATCTGCCAAGGATGT R: ACTCGGAGCGTCGATTTCTG	191	61°C	94.5	0.991

^a^: F-forward primer; R-reverse primer; Sequences are from transcriptome data of *P*. *donghaiense* sequenced using illumina (Supplemental information).

For normalizing these genes’ expression levels for comparison across different samples, *calmodulin* (*Pdcalm*) was selected as the reference gene because of its proven stability [33]. qRT-PCR amplification protocol was 1 cycle at 98°C for 3 min, 40 cycles of 98°C for 15 s and 58°C for 1 min. Gene expression levels under the experimental conditions relative to that in the control (L1 condition) was calculated using the ΔΔCt method [34,35], 2^-ΔΔCt^ = 2^-{[Ct(target)-Ct(calm)]sample-[Ct(target)-Ct(calm)]control}^.

Statistical analyses were performed using SPSS 13.0. for evaluating the statistical significance of difference between different culture conditions. Specifically, the differences between mean values from experimental groups and the control group (nitrate-replete) were determined by Student’s two tailed t-tests, and *p* < 0.05 was considered threshold of statistical significance.

## Results

### Growth on different N sources

**Fig 1:**
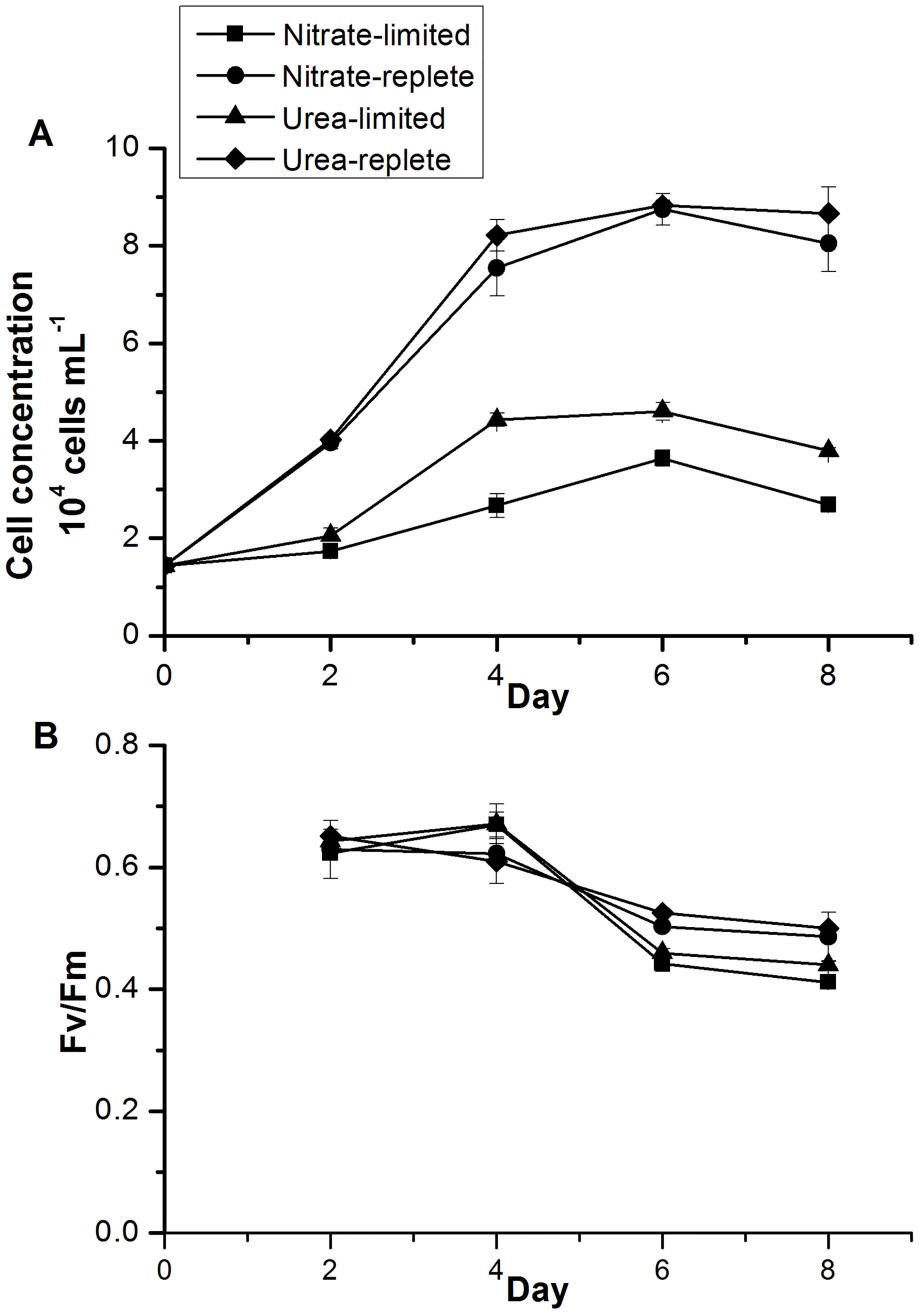
Growth curves (A) and maximum photochemical efficiency of PSII (*F*v/*F*m; B) in the *Prorocentrum donghaiense* cultures under the N-replete and N-deprived conditions in the 8-day experimental period. Shown are means ± standard deviations (error bars) from the triplicated cultures.

Growth difference was recorded between the N-limited and N-replete groups. As shown in Fig 1A, although initial cell densities in the four N treatment groups were similar, cell concentrations under N-limited and -replete conditions started to diverge within two days. The nitrate-replete and urea-replete groups exhibited a similar growth trend, showing exponential growth within the first 4 days, with the average growth rates of 0.41 day^−1^ and 0.44 day^−1^, respectively, followed by a stationary phase reaching their maximum cell concentrations of ∼ 8.7 × 10^4^ and ∼8.8 × 10^4^ cells mL^–1^, respectively throughout the experimental period. However, in the urea-limited group, maximum cell concentration was much lower, at ∼ 4.4 × 10^4^ cells mL^–1^, with an average growth rate of 0.19 day^−1^ during the exponential growth period; In the nitrate-limited group, cell concentrations maintained a slow exponential growth from 0 to 6 days with an average growth rate of 0.15 day^−1^ and a maximum cell concentration of ∼ 3.6 × 10^4^ cells mL^–1^.

### Photochemical efficiency of PSII

As shown in Fig 1B, Fv/Fm ratio, which characterizes the photochemical efficiency of PSII, showed no significant differences between the two N-replete and the two N-limited conditions during the experimental period. Between the N-replete and the N-limited groups, no significant difference was observed on day 2 and 4, but subsequently the ratio in the two N-limited groups declined sharply to lower levels than the N-replete groups (Fig 1B).

### N concentration in the medium and content in the cells

As shown in Table 2, nitrate concentration in the medium of the nitrate-replete group decreased dramatically while cell populations grew in the 8-day experimental period. Based on the cell concentrations, these gave an approximate uptake rate of 3.57 pmol N cell^-1^ d^-1^. In the nitrate-limited group, nitrate concentration in the growth medium decreased to undetectable levels after two days. Based on data on the first two days, the uptake rate was approximately 1.49 pmol N cell^-1^ d^-1^. In contrast, urea concentration in the medium of the urea-replete group declined gradually throughout the experimental period, and the average urea-N uptake rate was estimated as 3.3 pmol N cell^-1^ d^-1^. Different from the nitrate-limited group, urea in the urea-limited group was not exhausted until after six days (Table 2). Based on the cell concentrations, the average urea-N uptake rate was 1.34 pmol N cell^-1^ d^-1^.

**Table 2:** N-nutrient concentrations in the media in the first 6 days of experimental period.

Day	Nitrate or Urea (μM)			
	0	2	4	6
Nitrate-replete	953.16	890.23 ± 32	636.96 ± 68	494.94 ± 36
Nitrate-limited	17.27 ± 1.9	8.21 ± 1.6	N/D	N/D
Urea-replete	743.35	721.56 ± 7.3	653.17 ± 12	607.73 ± 67
Urea-limited	18.15 ± 0.63	14.19 ± 0.87	7.83 ± 1.3	2.39 ± 0.11

Data shown are means ± standard deviations from the triplicated cultures

The measurements of CHN elements in two consecutive days in the late exponential phase showed that cellular N contents were similar, around 12.24–12.67 pg N cell^-1^, between the nitrate-limited and the urea-limited group, giving a C:N ratio of 21 (Table 3). In the two N-replete groups, cellular N content increased slightly from the 4^th^ to 6^th^ day and N content was higher in the urea-replete group (27.77 pg N cell^-1^ and 29.10 pg N cell^-1^) than the nitrate-replete group (20.74 pg N cell^-1^ and 21.07 pg N cell^-1^). Cellular C content was higher in the N-limited group than the N-replete group (Table 3). Accordingly, C:N ratios in the nitrate-replete group were 9 (4^th^ day) to 10 (6^th^ day), in comparison to 7 in the urea-replete group, whereas that in the N-limited cultures was consistently 21 (Table 3).

**Table 3:** Cellular nitrogen and carbon contents of *P*. *donghaiense* grown under the N-replete and N-deprived conditions.

Day	Nitrogen supplies	pg N cell^-1^	pg C cell^-1^	C:N ratio
4	Nitrate-limited	12.61 ± 1.52	261.73 ± 4.48	21
	Nitrate-replete	20.74 ± 2.24	196.17 ± 4.04	9
	Urea-limited	12.42 ± 2.01	260.58 ± 5.41	21
	Urea-replete	27.77 ± 1.26	190.50 ± 4.04	7
6	Nitrate-limited	12.67 ± 1.24	267.23 ± 5.26	21
	Nitrate-replete	21.07 ± 2.34	208.09 ± 3.91	10
	Urea-limited	12.24 ± 1.01	264.02 ± 5.18	21
	Urea-replete	29.10 ± 2.87	213.31 ± 4.21	7

Data shown are means ± standard deviations from the triplicated culture

### Total RNA and N-related gene transcript abundances

The total RNA content in exponential cells showed significant differences between N-replete and -limited treatment groups. In the nitrate- and urea-limited groups it was 0.154 ± 0.04 and 0.141 ± 0.04 pg cell^-1^, respectively; 0.704 ± 0.10 pg cell^-1^ in the nitrate-replete and 0.778 ± 0.11 pg cell^-1^ in the urea-replete groups. As shown in Table 1, nearly identical efficiencies (94.5%–96.1%) were achieved for the primers used, the correlation coefficient (R^2^) ranged from 0.984 to 0.999 for the standards.

**Fig 2:**
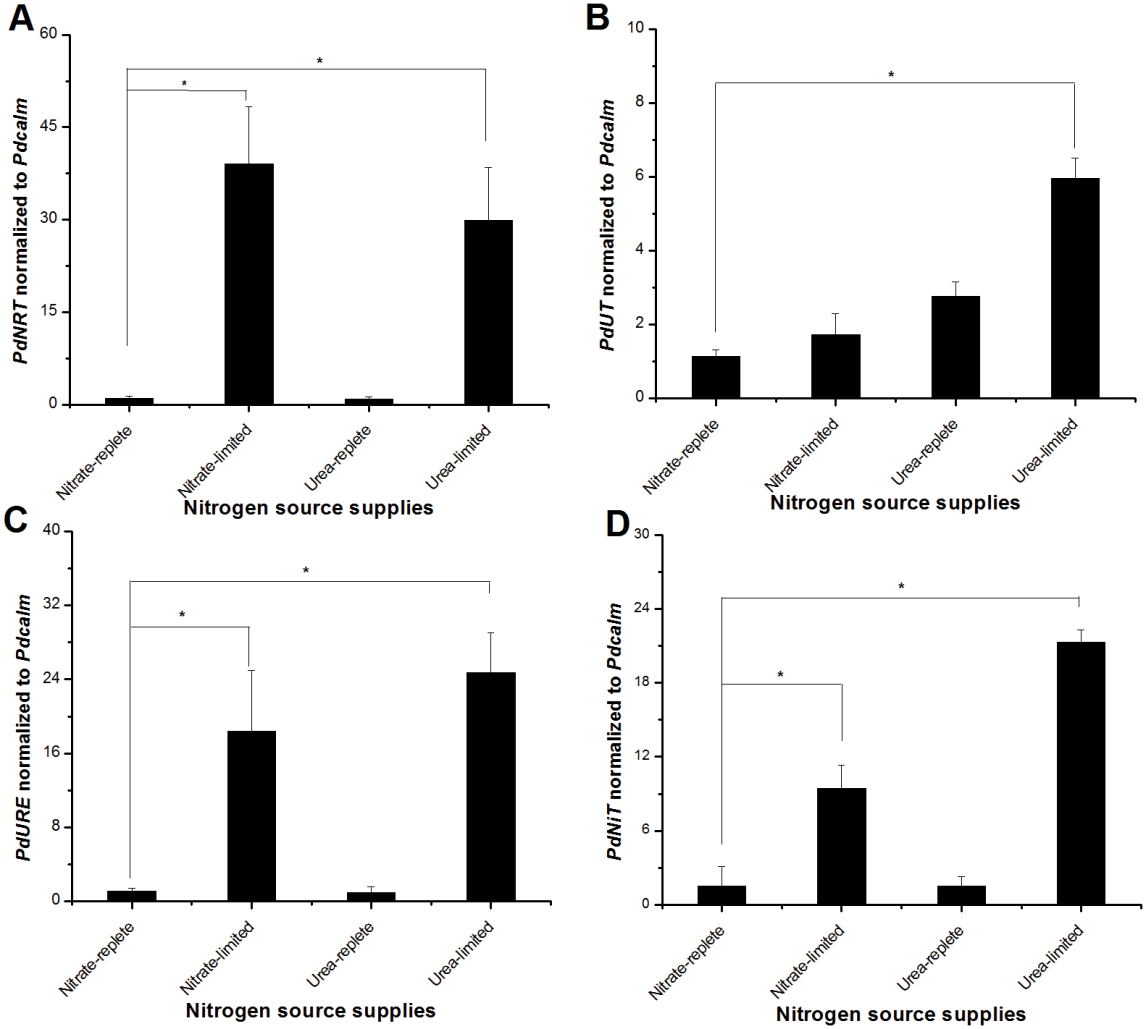
Transcriptional levels of N transporter and assimilation genes normalized to *calmodulin* (*Pdcalm*) in *Prorocentrum donghaiense* grown under nitrogen replete and deprived conditions. (A) Nitrate transporter (*PdNRT*). (B) Urea transporter (*PdUT*). (C) Urease (*PdURE*). (D) High-affinity nickel transporter (*PdNiT*). Error bars indicate ± SD of biological triplicates. Significant differences (*p* < 0.05) between experiment groups and control (nitrate-replete) are indicated by an asterisk (*).

Nitrate transporter transcript abundance normalized to the reference gene *calmodulin* (*calm*) was significantly higher under N-limited than N-replete conditions (Fig 2A). The average expression level of *PdNRT* in the nitrate-limited and urea-limited groups were about 38-fold (*p* < 0.05) and 28-fold (*p* < 0.05) higher, respectively, than the control group (nitrate-replete cultures). The urea-replete group showed a slightly lower expression level than did the control group, but not significantly. These results showed remarkable induction of *PdNRT* by N stress, both nitrate limitation and urea limitation.

As shown in Fig 2B, *PdUT* expression levels in the urea-limited group was about 5-fold higher than the control group. In contrast, *PdUT* relative expression level in the urea-replete group was only 1.5-fold higher than in control group; and the expression level in the nitrate-limited group showed no significant difference compared with control. These results indicated that *PdUT* expression more specifically responded to limitation in urea availability than to limitation of other forms of N.

Urease and high-affinity nickel transporter transcript abundances relative to *calm* showed a clear up-regulation under N-limitation regardless of if it was in nitrate culture or urea culture (Fig 2C and 2D). The average expression levels of *PdURE* under nitrate-limited and urea-limited groups were about 18-fold (*p* < 0.05) and 24-fold (*p* < 0.05) higher, respectively, than the cultures in nitrate- or urea-replete media. No significant difference was found in *PdURE* expression between the urea-replete group and the control group (nitrate-replete condition). A similar trend was noticed for *PdNiT*, the relative transcript abundance of which was about 8-fold (*p* < 0.05) and 22-fold (*p* < 0.05) higher in the nitrate-limited and urea-limited groups, respectively, than the nitrate-replete group as control, but no significant difference was detected between nitrate and urea-replete cultures (Fig 2D).

## Discussion

### High efficiency to utilize urea in *P*. *donghaiense*

Many phytoplankton species have been shown to be able to utilize DON. Dinoflagellates, in particular, seem to be efficient consumers of organic N [36,37]. The ability to utilize urea is often invoked to explain dinoflagellates dominant in eutrophic areas [38]. Moreover, it has been postulated that rising urea concentrations in the environment may trigger dinoflagellate blooms [39]. *Prorocentrum minimum* blooms in the Neuse River estuary were attributed to its high affinity for urea [37]; and in *L*. *polyedrum*, ambient urea was found to contribute a relatively large fraction of N demand [9]. Hu et al (2014) have reported that *P*. *donghaiense* grew well on various forms of DIN (NO_3_^2-^ and NH_4_^+^) and DON (urea, glutamic acid, etc) when they were supplied as the sole source of N; furthermore, maximum specific growth rates were 2-fold higher in cultures supplied with urea [40].

Similar to these previous studies, the present study showed that *P*. *donghaiense* has a strong ability to utilize urea at higher growth efficiency than on nitrate as sole N source. The urea-replete cultures exhibited a slightly higher growth rate and maximum biomass than nitrate-replete culture. The difference can be due to the actual higher N atom molar concentration in the urea medium when provided at equal molecule molar concentration as nitrate, but because both nutrients were provided at saturated concentrations (~ 880 μM), the likelihood was small, as verified by the fact that substantial amount of nitrate or urea remained in the stationary growth phase (Table 2). As a major nutrient, the switch of available forms of N can potentially affect various cellular activities including photosynthesis. In the present study, the *F*v/*F*m ratios of *P*. *donghaiense* exhibited no significant difference between urea and nitrate groups (Fig 1B), indicating that photosynthetic efficiency of *P*. *donghaiense* was not compromised by replacement of nitrate by urea although urea utilization involves transport of nickel and action of uease. In fact, the higher growth efficiency on urea than on nitrate is probably in part because nitrate needs to undergo a two- reduction process (from nitrate to nitrite then to ammonium). This may explain why blooming populations of dinoflagellates tend to have higher uptake rates for urea and amino acids than for nitrate [1].

Under N-replete conditions, although N concentrations in the medium decreased dramatically from the 4^th^ to 6^th^ day, cellular N contents stayed relatively stable (Tables 2 and 3). This indicated that the remaining N-nutrient was still enough to support growth. Although there was higher N content in urea-replete group than in the nitrate-replete group, C content was similar between these two groups. Accordingly, C:N ratio was lower in the urea replete group (7:1) than in the nitrate replete group, (9:1 to 10:1, Table 3). It is worth noting that C:N ratio 7 is close to the Redfield C:N ratio = 106:16 [41]. Because the change in C:N ratio has important biogeochemical implications, whether urea utilization would cause a lower C:N ratio in other species of dinoflagellates and phytoplankton should be investigated further in the future.

### Impacts of nitrogen deficiency on cell growth and C:N ratio

As a major nutrient, nitrogen is essential for cell growth, proliferation, and major metabolic activities. It is thus not surprising that growth was suppressed by N limitation, in both the case of nitrate and urea. As an indicator of photosynthetic status, studies have reported that the *F*v/*F*m ratio decreases under nutrient stress [42]. The descent of *F*v/*F*m ratio has been reported in green algae, *Dunaliella tertiolecta* under nitrogen limitation [43], as well as in cyanobacterium, *Synechococcus* spp. [44]. In the present study, the Fv/Fm ratio of *P*. *donghaiense* showed a substantial reduction in both the nitrate- and the urea-limited groups after 4 days (Fig 1B). This indicates that as in other phytoplankton, the photosynthetic efficiency of *P*. *donghaiense* was compromised by N deficiency.

Although the medium in the nitrate-limited cultures became depleted of nitrate (undetectable) as early as day 4 whereas that in the urea-limited cultures still had limited amount of urea (Table 2), cellular N content was maintained around 12 pg cell^-1^ in both groups from day 4 to day 6. This suggests that *P*. *donghaiense* needs to maintain a minimum cellular N content in face of ambient N depletion. To achieve this, this species seemed to stop its population growth under N deficiency. Importantly, such stable cellular N stores may confer advantages in habitats with a fluctuating N supply [45,46]. The increase in cellular C content and C:N ratio indicates continued photosynthetic C fixation with stalled cell division under N deficiency. The stable N content and increased C content may enable the cells to restore cell division rapidly upon resupply of N. It is noteworthy that phosphate limitation also allows continuation of photosynthetic carbon fixation and cell growth, as demonstrated in the dinoflagellate *Amphidinium carterae* [47]. Our results, while lending support to the widely recognized notion that the C:N stoichiometric ratio can be species specific and vary with environmental nutrient profiles, further provide insights into the mechanism, showing that the increase of cellular C content and decrease of cellular N content cause the shift of the C:N ratio, at least in the case of *P*. *donghaiense* (Table 3).

### Transcriptional responses to nitrate- and urea-limitation

Although dinoflagellates regulate only a small fraction of genes at the transcriptional level [2], transcriptional regulation of nutrient uptake and metabolism genes seem to be not uncommon [1,24–26]. Besides findings of transcriptional response of alkaline phosphatase to phosphate limitation, N stress has been shown to induce transcriptional responses in dinoflagellates, which may act to modulate N metabolism according to ambient N status [1]. We examined responses of four genes in *P*. *donghaiense*, which regulate the transport and assimilation of N. Of these, nitrate transporter (*PdNRT*) was expressed at a low level under nitrate- or urea-replete conditions, but exhibited very strong up-regulation under limitation of either nitrate or urea. This suggests that *PdNRT* is a high-affinity type of nitrate transporter and it responds to general N stress irrespective of the form of N to which the cells has previously been exposed. Similar up-regulation of nitrate transporter under nitrate limitation has been reported in the toxic dinoflagellate *Karenia brevi*s, the common diatom *Phaeodactylum tricornutum*, the prymnesiophyte *Emiliania huxleyi*, the pelagophyte *Aureococcus anophagefferens* and the prasinophyte *Micromonas* [26, 48–52]. Although in the diatom *Cylindrotheca fusiformis* induction of nitrate transporter transcription under urea has been deemed as a N-starvation response, this response to urea decline and limitation has not been reported in phytoplankton yet [53]. Urea transporter gene (*PdUT*), in contrast to nitrate transporter gene, appeared to be constitutively expressed at some levels, and was up-regulated under either nitrate- or urea-limitation, but was up-regulated at a much higher amplitude by urea-limitation. This indicates that *PdUT* expression responds more strongly to urea decrease. However, urease (*PdURE* gene), the enzymes catalyzing the breakdown of urea to ammonium and carbonate, and high-affinity transporter of Ni (*PdNiT* gene), the metal active center of urease, showed similar remarkable up-regulation under either nitrate or urea limitation as did *PdNRT*. These results suggest that while *P*. *donghaiense* cells are constantly ready to transport urea, the molecular machinery for assimilation is activated more strongly when external source of N is limited. Interestingly, as previously reported, dinoflagellates can store both urea and nitrate in the cell [54,55].

Consistent with the finding in the present study, many previous studies on various algae have shown that expression of the urea transporter gene is higher in N-limited than N-replete conditions. For example, an active urea transporter existing in the transcriptome of the haptophyte *Prymnesium parvum*, showed a higher expression level under N-limited condition than N-replete condition [56]. In *A*. *anophagefferens*, multiple putative transporter genes were shown to be induced by their substrate or N-limited treatments, though some of them seemed to be substrate- specific [57,58]. Like nitrate transporters, the urea transporter gene in this species was previously shown to be expressed at a higher level under N-deficiency than under N-sufficient conditions, and at a higher level in cultures grown in urea than those grown in other common N sources, indicating substrate-specific affinity [52]. These results also agree with findings on yeast and vascular plant urea transporter genes, which are induced by urea and specific to urea transport [59,60].

It is intriguing to note that *PdURE* was highly induced not only by urea stress but also by nitrate-limitation (Fig 2C). However, this is not unprecedented in algae. A study on *P*. *parvum* showed that urease gene was highly up-regulated when cells were nitrate limited compared to the replete treatment [56]. Similarly, in *Prochlorococcus*, urease gene was up-regulated during N deprivation [61]. Likewise, urease activity could be regulated by general nitrogen sources and inducible by these N deficiencies. In the dinoflagellate *P*. *minimum* as well as other phytoplankton, urease activity was expressed regardless of the chemical form of N [7]. *Karlodinium veneficum* exhibited a lower urease activity in cultures grown with ammonium as sole N source than in those grown with nitrate or with urea [62]. In *Alexandrium fundyense*, there was no detectable urease activity in nitrate treatment compared with maximum urease activity in urea treatment and N-deficiency [36]. In *Heterocapsa triquetra*, cultures grown on ammonium showed a higher urease activity than those on urea or nitrate [62]. The multi-level and substrate-dependent regulation on urease is probably due to the fact that it is related to not only internal recycling of ‘old’ N (product of purine metabolism or amino acid) but also producing ‘new’ N available from the ambient environment containing urea, purines, and other forms of organic N [63,64].

It is no surprise to observe a similar expression and induction pattern in *PdNiT* as in *PdURE* because nickel is a co-factor of urease. Ni-dependence of urea metabolism has been confirmed in some dinoflagellates, such as *A*. *catenella* and *A*. *fundyense* [37]. In turn Ni uptake rates can influence N nutrition status, as reported in diatom *Thalassiosira weissflogii*, in which growth rate experiment showed that low urease activity in cells was caused by Ni limitation [65]. The analogous Ni-N co-limitation has also been found in the cyanobacteria, *Synechococcus* [66]. To date, many studies have indicated that Ni plays a significant role in nitrogen metabolism. In fact, a study on cyanobacteria showed that *ntcA*, encoding cAMP receptor protein family, was a N-sensing regulator for modulating Ni uptake [66]. Whether a similar regulator exists in dinoflagellates warrants further investigation.

## Conclusion

In this study, we examined impacts of urea utilization and N deficiency on physiology and molecular regulatory mechanisms with which *P*. *donghaiense* responds to urea and general N variability. The coherent physiological and molecular results reveal the ability of *P*. *donghaiense* to utilize urea, even more efficiently than to utilize nitrate, yielding higher biomass and cellular N content. *P*. *donghaiense*’s ability to adapt to variable N availability may contribute to its ability to initiate and sustain blooms in a dynamic coastal environment. Nitrate or urea limitation resulted in the increased expression of nitrate transporter, urea transporter, urease and high affinity Ni transporter genes which are associated with processing extracellular N or reallocating intracellular N. The distinct C:N ratio (7:1) in urea-grown cultures and transcriptional responses of nitrate and urea transporters to N status suggest that they can potentially be used as tools with which to assess the N-nutrient status and hence the potential contribution of urea to the occurrence and maintenance of *P*. *donghaiense* blooms. Whether the findings in *P*. *donghaiense* apply to other dinoflagellates or even other groups of phytoplankton should be further investigated in the future.

## Supporting information

S1 File FASTA files of *Prorocentrum donghaiense* gene sequences for urease (PdURE), urea active transporter (PdUT), nitrate transporter (PdNRT), and high-affinity nickel transport protein (PdNiT). (ZIP)
