# Correction: Utilization of urea and expression profiles of related genes in the dinoflagellate *Prorocentrum donghaiense*

**DOI:** 10.1371/journal.pone.0191521

**Published:** 2018-01-16

**Authors:** Xiaoli Jing, Senjie Lin, Huan Zhang, Claudia Koerting, Zhigang Yu

The following information is missing from the Funding section: This work was supported by the National Natural Science Foundation of China (No. 41521064).
